# “Gestaltomics”: Systems Biology Schemes for the Study of Neuropsychiatric Diseases

**DOI:** 10.3389/fphys.2017.00286

**Published:** 2017-05-09

**Authors:** Nora A. Gutierrez Najera, Osbaldo Resendis-Antonio, Humberto Nicolini

**Affiliations:** ^1^Instituto Nacional de Medicina GenómicaMexico City, Mexico; ^2^Human Systems Biology Laboratory, Coordinación de la Investigación Científica - Red de Apoyo a la Investigación, Instituto Nacional de Ciencias Médicas y Nutrición Salvador Zubirán, National Autonomous University of Mexico (UNAM)Mexico City, Mexico

**Keywords:** systems biology, psychiatry, lung cancer, diagnosis, omics

## Abstract

The integration of different sources of biological information about what defines a behavioral phenotype is difficult to unify in an entity that reflects the arithmetic sum of its individual parts. In this sense, the challenge of Systems Biology for understanding the “psychiatric phenotype” is to provide an improved vision of the shape of the phenotype as it is visualized by “Gestalt” psychology, whose fundamental axiom is that the observed phenotype (behavior or mental disorder) will be the result of the integrative composition of every part. Therefore, we propose the term “Gestaltomics” as a term from Systems Biology to integrate data coming from different sources of information (such as the genome, transcriptome, proteome, epigenome, metabolome, phenome, and microbiome). In addition to this biological complexity, the mind is integrated through multiple brain functions that receive and process complex information through channels and perception networks (i.e., sight, ear, smell, memory, and attention) that in turn are programmed by genes and influenced by environmental processes (epigenetic). Today, the approach of medical research in human diseases is to isolate one disease for study; however, the presence of an additional disease (co-morbidity) or more than one disease (multimorbidity) adds complexity to the study of these conditions. This review will present the challenge of integrating psychiatric disorders at different levels of information (Gestaltomics). The implications of increasing the level of complexity, for example, studying the co-morbidity with another disease such as cancer, will also be discussed.

## Introduction

According to the World Health Organization (WHO), the frequency of psychiatric diseases has been steadily increasing (World Health Organization, 2011). Furthermore, many patients do not fully respond to therapy. There is a currently limited knowledge on the pathophysiology of neuropsychiatric disorders, which in turn diminishes the ability to identify clinical biomarkers for the early diagnosis of patients at risk (Martins-de-Souza, 2014; Sethi and Brietzke, 2015).

The classical approach for psychiatric diagnosis includes an essential evaluation on the mental health of the patient, by means of an interview, to determine the presence of a series of signs and symptoms (Fatemi and Clayton, 2008). For instance, paranoid schizophrenia is diagnosed by the presence of delirium, hallucinations, self-inflicted injuries, personality disorders, lack of substance abuse, and the continuity of this clinical frame for more than 6 months. In addition, the Diagnostic Interview for Genetic Studies (DIGS) is widely used in the diagnosis of schizophrenia, validated for both USA and non-USA populations, along with additional sources of information such as the Family Interview for Genetic Studies (Contreras et al., 2009). In this case, laboratory studies such as urine drug screens or sleep-deprived electroencephalograms are used to exclude stimulant-induced psychosis or complex partial (temporal lobe) seizures (Lishman, 1987). A positive familiar history provides further support in the diagnosis of schizophrenia. Thus, the diagnostic process in psychiatry is analogous to other branches of medicine where personal and familiar history, physical examination, and laboratory tests constitute essential steps. Regardless, it is difficult to obtain an accurate description without careful and skillful probing during face-to-face interviews. However, this phenomenology can be interpreted under different theoretical frames of reference pertaining to the formulation of the case but not to diagnosis (Fatemi and Clayton, 2008).

Although the Diagnostic and Statistical Manual of Mental Disorders (DSM) is often useful in classical diagnoses, it is not designed to facilitate the development and integration of biomedical knowledge. Therefore, the National Institute of Mental Health has developed an alternative tool known as the research domain criteria (RDoc). This multidimensional approach utilizes units of information beyond clinical phenotypes, i.e., imaging, behavior, etc. Thus, a matrix is developed with constructs that can be related to different elements of information ranging from imaging to genetics (American Psychiatric Association).

The Human Genome Project, along with high throughput technologies, has increased the biological knowledge of several human illnesses. The genome sequencing and analyses of physiological states have further contributed to this purpose. However, the genome as a whole is difficult to interpret and in the case of several multi-factor diseases such as diabetes, cancer and neurological disorders, which often involve the function of a large number of genes, biological pathways, and environmental factors, can further convolute an assessment. Therefore, the combination of genomic information with a detailed molecular analysis will be important in the prediction, diagnosis and treatment of diseases, also allowing the understanding of initiation, progression, and prevalence of disease states (Williams et al., 2004; Shi et al., 2009). In this regard, metabolomics is the newest of the “omics” sciences; it provides a comprehensive approach to understanding the biochemical regulation of metabolic pathways and networks in a biological system. Metabolomics is able to complement the data from genomics, transcriptomics, and proteomics to provide a potentially systemic approach in the study of central nervous system (CNS) diseases (Weckwerth and Morgenthal, 2005). However, there are few currently available studies in neuroscience regarding the data integration from different “omics” sciences.

Often, neuropsychiatric diseases are biologically difficult to define partly because the brain is more difficult to access than other parts of the body. Moreover, research in psychiatry is compounded by the complexity of the brain and the heterogeneity of phenotypes in psychiatric disorders. Brain imaging, genotyping, and immune system testing are important approaches in understanding the biology of psychiatric illness. The advances in technology have made possible the analysis of whole units of cellular components. Regardless, the study of protein and metabolic function in the CNS is made difficult because of intricate cellular heterogeneity with a complex neuronal morphology that includes cellular compartments such as neural dendrites, postsynaptic dendritic spines, axons and presynaptic terminals. Another factor contributing to the difficulty in studying the metabolome of CNS in humans is the limited access to either tissue or fluids, such as cerebrospinal fluid (CSF), in order to study molecular alterations in psychiatric disorders. Due to ethical considerations, it is often preferable to analyze peripheral samples such as plasma, serum, leukocytes and platelets, which are more easily available (Hayashi-Takagi et al., 2014). An “omics” approach has the potential to accelerate the discovery of markers for CNS diseases (Niculescu et al., 2015a). As an example, there is already the use of Systems Biology in the analysis of data from several “omics” technologies, such as proteomics, improving the discovery of pathophysiological mechanisms and biomarkers for brain injuries that could lead to Alzheimer's and Parkinson diseases (Abou-Abbass et al., 2016; Jaber et al., 2016a,b).

The tendency today is to integrate the data from clinical and “omics” studies to obtain a final behavioral phenotype (phenome) (Williams et al., 2004; Monteith et al., 2015; Sethi and Brietzke, 2015). Genome-wide association (GWA) studies with metabolic measurements have shown that genetic variation in metabolic enzymes and transporters lead to concentration changes of their respective metabolites (Suhre et al., 2011; Krumsiek et al., 2012). The main goal of these studies is to identify new interactions between genomic and metabolic systems, yielding valuable insight for basic research and clinical application. The analysis of metabolic data is often the result of several processes where a substance can be identified as unique in the sample but the specific process from which it was derived is unknown. This concept is similar to the identification of a fingerprint: each one is identifiable as unique, but it needs to be registered in a database, that way we know who owns that print. The association with genetics provides evidence of the metabolic pathway wherein such a metabolite is involved and the process from which it originates (Suhre et al., 2011; Krumsiek et al., 2012).

From the point of view of Gestalt psychology, the first biological response is organized as units or structures, these organized units or “gestalten” correspond to the exchange of information and interactions between environmental stimuli and the individual. The resulting “gestalten” are different than the sum of their factors so “there is a tendency not only to perceive the gestalten but also to complete and reorganize them according to biological principles, which will vary in the different levels of maturation or growth and the pathological states” (Bender, 1938). Currently, the approach based on systems biology methods is the most suitable for data integration from different levels of information (genome, transcriptome, proteome, epigenome, metabolome, phenome, and microbiome), in order to unify and reorganize these “gestalten” (organized units of biological or clinical data) in an integrated view of the psychiatric patient. Therefore, “Gestaltomics” is an integrated view of different levels of information ranging from clinical to “omics” data, proposing the diagnosis of neuropsychiatric diseases. Early diagnosis of these disorders could reduce the risk of developing chronic diseases such as obesity, diabetes, cancer, etc. Past research has proposed that affective disturbances involving mood alterations, anxiety, and irritability may be signals of medical conditions along with psychiatric diseases (Cosci et al., 2015). In this regard, depressive symptoms are of first occurrence in approximately 38–45% of pancreatic carcinoma cases and symptoms of a major depressive illness may precede the diagnosis of lung cancer (Jacobsson and Ottosson, 1971; Hughes, 1986). These studies conclude that the development of psychiatric illness early in the course of a medical condition could affect the prognosis and therapy for patients diagnosed with the same medical disease.

On the other hand, addictive disorders are a class of chronic, relapsing mental disorders that often result in death. In fact, tobacco dependence is related with a higher risk for disease and premature death because of its association with several major health problems including respiratory and cardiovascular diseases, and cancer. There is a current initiative to test the Smokescreen genotyping array, a research tool for the significant advance in understanding addiction and the development of predictive models for personalized treatment strategies. This array includes markers related to addiction and, interestingly, it also has an additional set of comorbidity markers for lung cancer and other psychiatric disorders (Baurley et al., 2016).

Therefore, understanding the molecular factors contributing to psychiatric illness and identifying new biomarkers is essential in the proposal of alternative tools for diagnosis, prognosis, screening, or therapeutic targets. This manuscript describes some examples on the current knowledge of the “omics” field in three psychiatric conditions and their correlation with complex diseases, mainly cancer.

### “Omics” technologies applied in schizophrenia

Schizophrenia was described by Emil Kraepelin as “dementia praecox, separated from manic-depressive psychosis” (Kraepelin, 1893). The current criteria for schizophrenia diagnosis has been compiled from years of empirical testing and recorded in the Diagnostic and Statistical Manual, 5th edition (DSM-5). The existence of different types of schizophrenia has been proposed, each one with its own phenotype and genotype. Most research has been focused on loci in chromosomes 6, 8, 13, and 22. Of these chromosomes, chromosome 22 calls for attention since it contains the *comt* (catechol *o*-methyltransferase) gene, involved in dopamine metabolism. Therefore, individuals with a particular *comt* genotype (e.g., val/val allele) are at risk developing schizophrenia (Combs et al., 2012). Research conducted on samples from schizophrenia patients, both peripheral and postmortem brain samples, revealed a correlation, although low, in the results obtained from peripheral samples (blood, plasma, serum, and platelets) compared to CNS samples (CFS, prefrontal cortex and other brain tissues). In one of these studies using DNA microarrays, postmortem analyses detected 177 genes in schizophrenia related brains. From these genes, only 6 correlated with the obtained blood results (Glatt et al., 2005). In another study, half of the genes found related to schizophrenia in the prefrontal cortex were also found in blood from the same patients (Sullivan et al., 2006). The hypomethylation of *st6galnac1* in the blood and brain of schizophrenia patients has been previously reported (Dempster et al., 2011). Allele copy number variations (CNVs) seems to be the most relevant risk factor for schizophrenia, and the 15q11.2 (BP1-BP2) deletion confers the risk for developing schizophrenia (Stefansson et al., 2013). Using metabolomics, an increment in free fatty acids and ceramide in blood and brain samples was observed (Schwarz et al., 2008). Proteomics experiments using SELDI-TOF MS showed that the ApoA1 protein was downregulated in CFS and blood (red blood cells; RBC) (Huang et al., 2007). On the other hand, current advances in schizophrenia physiopathology research and the molecular effects of anti-psychotic drugs have made clear the need of biomarkers for this disease. Metabolomics techniques are not only useful in this purpose but also in monitoring the effect of these types of drugs in psychiatric patients.

A metabolomics study of serum using mass spectrometry (MS) reported 20 metabolites in patients with schizophrenia whose levels were modified when compared with the controls. These metabolites include citrate, palmitic acid, allantoin, and mio-inositol (Xuan et al., 2011). He et al. (2012) performed a nuclear magnetic resonance (NMR) study in the plasma from schizophrenia patients. In this study, the patients were diagnosed before starting the treatment. There was also a group of subjects under medication. Both groups were compared to the control group (no schizophrenia), identifying different metabolites from to the study performed by Xuan.

### “Omics” technologies applied in autism

Autism spectrum disorders (ASDs) are highly hereditary and genomic studies have revealed that a substantial proportion of ASD risk resides in rare variations ranging from chromosome abnormalities (CNV) to single-nucleotide variations (SNV). These studies highlight a striking degree of genetic heterogeneity, implicating both de novo germline mutation and rare inherited ASD variations (Pinto et al., 2014). *De novo* CNVs are observed in 5–10% of screened ASD-affected individuals, and after further follow-up studies, some of them have been shown to alter high-risk genes. De novo or transmitted CNVs, such as 15q11.2–q13 duplications of the affected region in Prader-Willi and Angelman syndromes, the 16p11.2 deletion, 16p11.2 duplication, and X-linked deletions, including the PTCHD1-PTCHD1AS locus, have also been found to contribute to this risk (Stefansson et al., 2013). Exome and whole-genome sequencing studies have estimated at least another ~6% contribution to ASD and an additional 5% conferred by rare inherited recessive or X-linked loss-of-function (LoF) SNVs (Pinto et al., 2014 and references therein). A genetic overlap between ASD and other neuropsychiatric conditions has been increasingly recognized. Informative studies on the metabolome of ASD individuals showed alterations in the levels of amino acids in plasma, platelets, urine and CSF (Ming et al., 2012). Further, it has been reported that the neurotransmitter and hormone metabolism of serotonin, catecholamines, melatonin, oxytocin, GABA, and endorphins, for example, are altered. In a case-control study, changes in the levels of succinate and glycolate in urine were observed (Emond et al., 2013). Therefore, alterations in metabolism are common features of ASD. In this regard, gut microbiota has important effects in the development of behavioral symptoms relevant to ASD and other neurodevelopmental disorders in a mouse model (Hsiao et al., 2013).

### “Omics” technologies applied in suicide

In the area of mental health, suicide is a particular prevention priority as it accounts for an estimated 804,000 deaths in 2012 (World Health Organization, 2015b). An objective of WHO Mental Health Action Plan calls for a 10% reduction in the rate of suicide by 2020. Men are four times more likely to commit suicide than women. However, women make more nonfatal suicide attempts than men. There are several factors involved in suicide and suicide attempts, the most important of which is having a psychiatric disorder. More than 90% of suicides have a diagnosable psychiatric disorder at the time of death, mood disorders being the most common (Fatemi and Clayton, 2008). The origin of suicidal behavior is multifactorial and includes genetic, biological, and psychosocial factors. The *slc6a4* gene has been associated with suicide but only in women (Gaysina et al., 2006). The gene *comt* has been related to suicide in both women and men, but the degree of association differs between genders (Kia-Keating et al., 2007). GWAs have found gene markers for suicidal ideation such as polymorphisms rs11628713 and rs109030324 of genes *papln* and *il28ra*, respectively (Laje et al., 2009). A study addressing the relationship between genotype and brain transcriptome reported that the GABA A receptor gamma 2 (*gabrg2*) had lower postmortem expression in the brains of suicide cases and was thus associated with suicide (Yin et al., 2016). Amongst the polygenes implicated with 590 suicide attempts (SA) were several associated with important development functions (cell adhesion/migration, small GTPase and receptor tyrosine kinase signaling), and 16 of these SA polygenes have previously been studied in suicidal behavior (*bdnf, cdh10, cdh12, cdh13, cdh9, creb1, dlk1, dlk2, efemp1, foxn3, il2, lsamp, ncam1, ngf, ntrk2, and tbc1d1*) (Sokolowski et al., 2016). A recent study sought biomarkers for suicidal ideation using functional genomics. The authors identified genes involved in neuronal connectivity and schizophrenia, and the biomarkers validated for suicidal behavior included a wide number of genes involved in neuronal activity and mood. The 76 biomarkers validated for suicidal behavior map to biological pathways involved with the immune and inflammatory response, mTOR signaling, and growth factor regulation. Further, other potential therapeutic targets or biomarkers for drugs known to mitigate suicidality were identified, such as omega-3 fatty acids, lithium, and clozapine. These biomarkers are also involved in psychological stress response and in programmed cell death (apoptosis) (Niculescu et al., 2015b). A proteomics study of prefrontal cortex tissues showed that alpha crystalline chain B (CRYAB), glial fibrillary acidic protein (GFAP), and manganese superoxide dismutase (SOD2) appear only in suicide victims (Schlicht et al., 2007). Despite the vast amount of information from suicide “omics,” it has not been possible to integrate the data to provide a “gestalt” view of the individual, allowing the prevention of this behavior and its outcome. Thus, the integration of this knowledge will provide new methods for the diagnosis and treatment of this complex behavior.

### Adding one level of complexity: comorbidity of cancer and psychiatric disorders

To impulse the advance toward a new era of precision medicine, in 2015 President Obama proposed a research initiative (www.whitehouse.gov/precisionmedicine). Precision medicine includes prevention and treatment strategies taking individual variability into account. This concept has been improved by the development of large-scale biological databases, powerful methods for characterizing patients, and computational tools for the analysis of large data sets. The proposed initiative has two main components: a near-term focus on cancer and a long-term aim to generate applicable knowledge for the whole range of health and disease (Collins and Varmus, 2015).

Cancer is a major public health problem and a challenge that needs to be solved by a multidisciplinary approach (World Health Organization, 2015a). Its appropriate control includes health care education to improve prevention and early detection programs; and optimizing diagnosis to determine specific treatment and provide palliative care improving the patients' quality of life (Mohar et al., 2009).

The need for psychiatric services in hospitals can be observed by the high prevalence of psychiatric disorders. In oncology hospitals, the prevalence of these disorders is approximately 50% (Citero Vde et al., 2003). A study evaluating the prevalence of psychiatric illness in cancer patients reported that 47% of cancer patients diagnosed with mental disorders, amongst them 85% with anxiety and depression, 8% with cerebral organic disorders, and 7% with personality disorders (Citero Vde et al., 2003). Another study reported this type of disorder in 11–21% of patients at the hospital (Razavi et al., 1990). Delirium is often found in patients at the general hospital. The prevalence is 25% in cancer patients, and 85% in terminally ill patients. Psychoses and cognitive impairment have demonstrated a key role in slowing down the progress of cancer treatment in these patients (Citero Vde et al., 2003). A psychiatric comorbidity between smoking and psychosis has severe effects in the morbi-mortality in those patients and results in an increased number of deaths by suicide. It has been estimated that schizophrenia patients are addicted to nicotine in 80% of cases compared to 22% in the healthy population (Brown, 2000). On the other hand, obsessive-compulsive disorder seems to be a protective pathology against nicotine addiction (Dell'Osso et al., 2015).

Cancer is a disease whose treatment has high personal, financial, and social costs. These factors influence the development of anxiety and depression disorders in the cancer patient. Even cancer treatments such as chemotherapy, which produces serious secondary effects, affect this condition. For instance, breast cancer comorbidity with depression is associated with a poorer quality of life, poor treatment adherence, impaired physical and cognitive function, and cancer progression or survival. Understanding depression etiology associated with breast cancer is a major concern. Depression in breast cancer patients is often the result of several contributing biological factors; amongst them are hormonal, inflammatory, and genetic mechanisms, and psychological factors such as bodily disfigurement and impaired sexual function. Genetic risk is important in the etiology of depression precipitated by medical conditions like cancer, which has been proposed as an environmental risk factor (Caspi et al., 2010). Smoking is one of the main risk factors within these environmental factors. In fact, the WHO global initiative for Framework Convention on Tobacco Control (Deland et al., 2003) is one of the first strategies for primary prevention of cancer, because tobacco is related to 16 different types of cancer and smoking is the cause of 71% of deaths due to lung cancer (2015). The knowledge from the biological, molecular, and clinical data could improve the outcome for this disease and help avoid the behavior increasing the susceptibility for cancer development. A broad research program to improve creative approaches to precision medicine, test them rigorously, and use them to build the evidence base needed to guide clinical practice is essential (Collins and Varmus, 2015). A clear example for this is the relation between smoking and lung cancer.

### Nicotine addiction and lung cancer

The clearest example of how a psychiatric disorder influences the development of cancer is the relation between smoking and lung cancer. Smoking is an addictive disorder and a major public health concern. It is the primary cause of death worldwide, as actively smoking causes different chronic diseases, several types of cancer, and respiratory and cardiovascular diseases (World Health Organization, 2008).

There is evidence presented in the 2014 Surgeon General's Report (US Health Department) modifying cancer care. The detrimental consequences of smoking in patients with cancer are mediated by the activation of tumorigenic pathways and physiological alterations, including the complications associated with cancer treatment and development of comorbidities. However, no cancer treatment has been proved more effective in cancer patients who smoke compared to non-smoking patients, neither are there any prognostic biomarkers for cancer patients who continue to smoke (US Department of Health, 2014).

If both processes share the same molecular basis, and therefore the same biological pathways, it is important to highlight the need to study psychiatric diseases along with other co-morbidities such as cancer. The neuronal acetylcholine nicotinic receptors (nAChRs), a protein family of pentameric ion channels regulated by ligands, are potential candidates. These receptors can mediate signal transmission through the synapse as well as release of several neurotransmitters. The receptor subtype in the brain is the α4β2 form. Some α4β2 receptors also contain subunit α5, which is regulatory, inactivating the receptor. Nicotine is an exogenous agonist of these receptors. Seconds after starting to smoke, nicotine produces a physical response. Recent studies show that nicotine, despite not being carcinogenic, promotes cell proliferation, metastasis, angiogenesis, and resistance to apoptosis (Warren et al., 2014, and references therein). These processes, mediated by nAChRs, may influence the effectiveness of anti-cancer treatment (chemotherapy, radiotherapy, or targeted therapy). The evidence indicates that smoker patients have lower survival rates than those patients giving up smoking before starting treatment; suggesting that nicotine supplemented for smoking cessation treatment reduces the response to anti-cancer drugs (Czyzykowski et al., 2016).

Nicotine and its metabolites activate nAChRs and β-adrenergic receptors that in turn activate several pathways, such as the Ras/Raf/MEK/MAPK and PI3K/Akt oncogenic pathways, and causing cross-activation of these pathways producing a tumor-promoting phenotype. Furthermore, nicotine and the activation of nAChRs decrease the therapeutic response to chemotherapy and radiotherapy both *in vitro* and *in vivo* (Dasgupta et al., 2006; Warren et al., 2010; Momi et al., 2012).

Genetic variations in nAChRs have been proposed as strong risk factors for nicotine dependence and susceptibility to lung cancer. GWAS involving human addictions in lung cancer patients have reported the same variants in the gene cluster *chrna5/a3/b4*, previously associated with nicotine dependence and lung cancer susceptibility (Wang et al., 2009). This gene cluster plays a key role in nicotine dependence, lung cancer and loss of lung function when the allele A of the polymorphism rs16969968 is present (Gabrielsen et al., 2013). Moreover, nicotine was suggested as an intermediary factor between variants at the *chrna5/a3/b4* region and lung cancer (Tseng et al., 2014). Although it was previously considered that rare non-synonymous variants in this region played a protective role, the variant rs56501756, encoding for R336C, confers a risk for nicotine dependence, lung cancer and other smoking-related diseases (Thorgeirsson et al., 2016).

Moreover, there is evidence that smoking cessation treatments are affected by genetics. The *chrna5/chrna3/chrnb4* cluster defines haplotypes of low, intermediate and high risk of cessation treatment failure, according to the presence of polymorphisms rs16969968 and rs680244 (Chen and Bierut, 2013). Therefore, the identification of smokers with different haplotypes implies the need for personalized smoking cessation treatments.

However, research is not limited to genetic data only; there is research on nicotine metabolism and genotype association as well. One example of this concerns the *cyp2a6* gene coding for P450 2A6, the major nicotine metabolizer enzyme. Genetic variations in the cytochrome *cyp2a6* gene contribute greatly to the observed differences in nicotine metabolism, thus influencing smoking habits in different populations (Park et al., 2016). Differences in nicotine metabolism and risk of nicotine addiction have been attributed to functional allelic variation in *cyp2a6* (Mwenifumbo and Tyndale, 2009; Al Koudsi and Tyndale, 2010). The meta-analysis of samples from the ENGAGE consortium proved the association between SNP's in this locus and the number of cigarettes smoked per day (Thorgeirsson et al., 2010). Further evidence on the association of *cyp2a6* with the number of cigarettes smoked per day and nicotine dependence is observed in the synergic effects of the *chrna5/chrna3/chrnb4* cluster and this gene, showing independent and additive effects of allelic risk for these two chromosomal regions in two phenotypes (Wassenaar et al., 2011).

Active smoking is an established critical factor for epigenetic modification. Methylation changes were detected studying the association of active smoking exposure with methylation patterns; amongst these studies were epigenome-wide association studies (EWASs) and gene-specific methylation studies (GSMSs) (Gao et al., 2015). At molecular level, epigenetic factors such as DNA methylation have been proposed as biomarkers for both psychiatric disease and cancer (Ai et al., 2012). The correlation between methylation in leukocytes from patients with Parkinson disease and in neurons from the same patient has been reported (Masliah et al., 2013). In breast cancer, methylation of the *bdnf* gene (brain-derived neurotrophic factor) has been studied in relation with depression in mastectomy patients (Kim et al., 2013; Kang et al., 2015). In fact, the onset of smoking has been associated with *bdnf*, a neurotrophin identified as a possible candidate gene (Tobacco Genetics Consortium, 2010).

### Systems biology and the challenges in understanding the underlying mechanisms of human behavior

Data-intensive science consists of three basic activities: capture, curation, and analysis. These phases raise a challenge in systems biology science. These challenges entail not only their size but also their increasing complexity. Curation and analysis become important after capturing data from several experiments. It includes storage, retrieval, dissemination, and data filtering and integration. Algorithms and software tools developed for the analysis of biological data also face the problem of scalability when data become larger. However, several big databases have been created around the world for the curation and analysis of biological data, and their data volume and performance are gradually improving. These databases include GeneBank and Gene expression omnibus (GEO) from NCBI (Altaf-Ul-Amin et al., 2014). Recently, other projects have been initiated such as ENCODE (Encyclopedia of DNA Elements), a project supported by an international collaboration of research groups funded by the National Human Genome Research Institute (NHGRI/NIH). ENCODE aids the biologist using human and/or animal genetic data to study disease with a comprehensive list of functional elements in the human genome, including elements that act at protein and RNA levels, and regulatory elements that control cells and the circumstances in which a gene is active. Further, global metabolomics are used for the identification of metabolic pathways altered due to disturbances in biological systems. The statistical analysis involves an extensive process that sometimes may lead to the identification of a very narrow range of metabolites as biomarkers. In this regard, The Human Metabolome Project, funded by Genome Canada, was launched in 2005. The purpose of the project is to facilitate metabolomics research by providing a linkage between the human metabolome and the human genome. The project mission is to identify, quantify, catalog and store all metabolites that can potentially be found in human tissues and biofluids at concentrations greater than one micromolar. These data are free to access through the Human Metabolome Database (www.hmdb.ca) (Wishart, 2007; Wishart et al., 2009, 2012). The application of metabolomics in cancer research has led to a renewed appreciation of metabolism in cancer development and progression. It has also led to the discovery of biomarkers and novel cancer-causing metabolites. However, with so many cancer-associated metabolites being identified, it is often difficult to associate these compounds with their respective cancer type. It is also challenging to track down the information on the specific pathways that particular metabolites, drugs or drug metabolites may be affecting (Wishart et al., 2016).

The ENIGMA Consortium is an initiative seeking to integrate genetics, genomics and brain imaging (http://enigma.ini.usc.edu); it is a global alliance of over 500 scientists spread across 200 institutions in 35 countries collectively analyzing brain imaging, clinical and genetic data. ENIGMA has grown to over 30 working groups studying 12 major brain diseases, pooling and comparing brain research data. In some of the largest neuroimaging studies to date, such as in schizophrenia and major depression, ENIGMA has found replicable disease effects that are consistent worldwide, as well as common factors that modulate disease effects in different populations (Thompson et al., 2015, 2016).

Systems biology is being used to analyze data from different levels of information in psychiatric disease. In a study of CNVs and SNVs in genes related to ASD, chromatin remodeling and transcription regulation were inferred on functional gene networks related to neuronal signaling, development synapse function, chromatin regulation, MAPK, and other signaling pathways (Pinto et al., 2014). Other studies in systems biology suggest that the interplay between sleep, stress, and neuropathologies emerge from genetic influences on gene expression and their collective organization through complex molecular networks relating to underlying sleep mechanisms, stress susceptibility and neuropsychiatric disorders (Jiang et al., 2015). In animal models, a systems biology study based on proteomic and metabolomic research developed a schematic model summarizing the most prominent molecular network findings in the Df(16)A± mouse (a model of the 22q11.2 deletion syndrome). Interestingly, the implicated pathways were linked to one of the proteomic candidates, O-Linked N-acetylglucosaminyltransferase (OGT1), a predicted miR-185 target and a new mechanism associated with 22q11DS, which may be linked to a cognitive dysfunction and an increased risk of developing schizophrenia (Wesseling et al., 2016).

**Figure 1:**
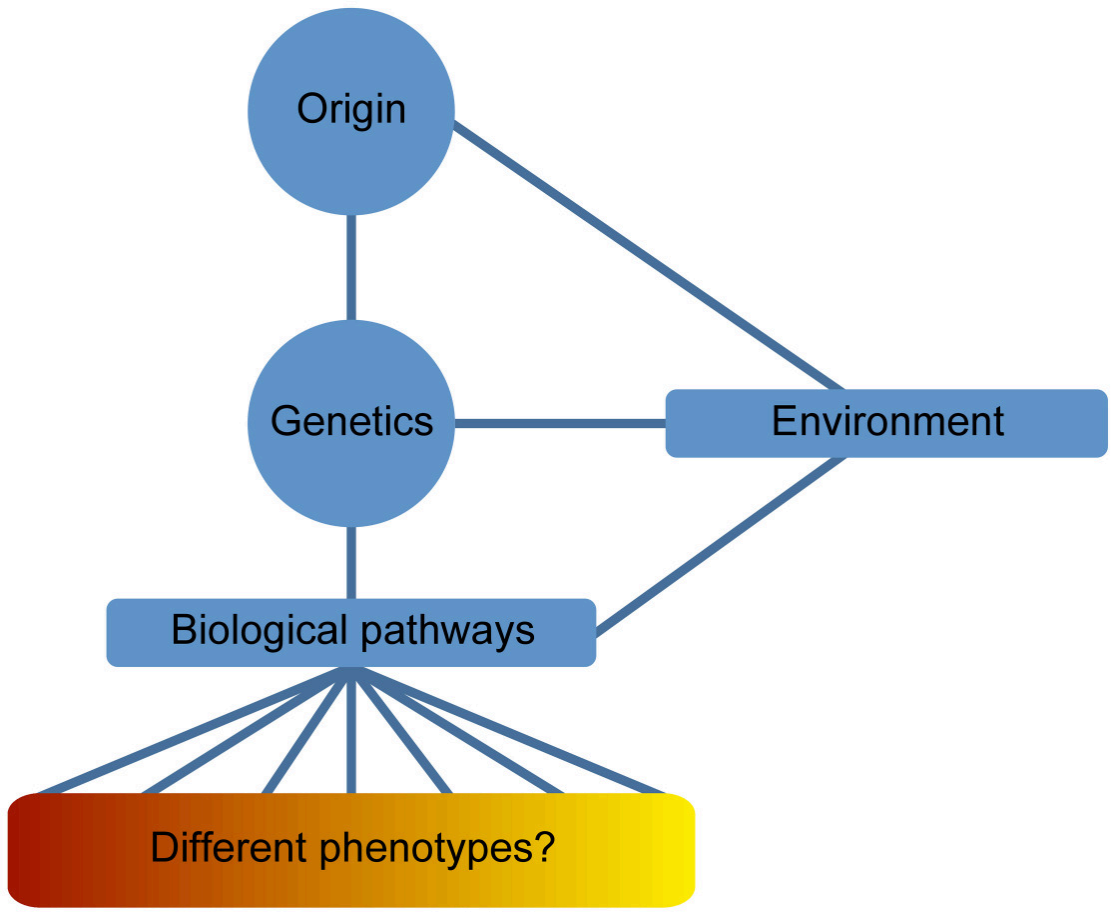
**Disease spectrum of Psychiatric illnesses**. The definition of neuropsychiatric phenotypes has been difficult to limit into a series of signs and symptoms overlapping the different psychiatric diseases. This issue is usually observed beyond clinical level; however, “omics” data have facilitated the contemplation of psychiatric illnesses as a disease spectrum of the brain. Genetics is an important component in the origin of the disease and the resulting phenotype is determined by several intermediate phenotypes derived from the influence of epigenetic factors (environmental stimuli).

An analysis comparing proteome and biological pathways and their involvement with different psychiatric illnesses showed molecular similarities across all major neuropsychiatric disorders. These results, analyzed by systems biology methods, proved an overlapping of pathways affecting protein expression in a similar manner in these disorders. This supports the hypothesis that major neuropsychiatric disorders represent a disease of the brain with a spectrum of phenotypes derived of the genotype and the effect of the environmental stimuli (Figure 1; Gottschalk et al., 2014).

One of the best efforts to materialize the integration of the phenome with the genome is exemplified by the Consortium for Neuropsychiatry phenomics, at the University Of California in LA (UCLA) (Bilder et al., 2009b). Besides making available a brain imaging database of healthy individuals and patients with neuropsychiatric disorders such as schizophrenia, bipolar disorder and attention deficit/hyperactivity disorder, it also provides bioinformatics tools to visualize and analyze these dataset in a “systematic study of phenotypes on a genome-wide scale,” including basic and clinical information (Poldrack et al., 2016). The concept of phenome is evolving to phenomics or “the discipline to enable the development and adoption of high-throughput and high-dimensional phenotyping” (Bilder et al., 2009a; Houle et al., 2010). The “phenomics” proposal of the Consortium of Neuropsychiatry includes an integrative vision of data in other complex biological systems and is already achieving that integrating vision (Bilder et al., 2013). We wish to convey this vision in medical practice, one that will also consider the socio-cultural issues and comorbidities of the patients. Of course, conveying the idea that “phenomics” applied to patient-centered medical practice will be “gestaltomics” in the near future.

Despite the efforts to integrate several networks of information, it has not been possible to personalize medicine through an integrative view of the individual through different levels of information; therefore, “gestaltomics” is an unifying vision of different sources of information through a systems biology approach that is not limited to a biological understanding of the disease and instead follows an old medical principle from Hippocrates “It is far more important to know what person the disease has than what disease the person has.” The onset of symptoms identify the clinical stage of the disease at the time of diagnosis. The disease can progress to mild, severe or fatal, i.e., “the spectrum of disease.” The disease process results in recovery, disability or death, which is the reason why it is important to identify the individuals at risk (The Center for Disease Control and Prevention, US Department of Health Human Services, 1992). The early screening of a high-risk group, such as smokers, during the subclinical stage of the disease could identify a difference in the development of a disease such as cancer or influence the outcome to this disease. These screenings could involve the analysis of blood and urine samples, which are easy to obtain. It could involve the genotyping of genes, such as the cluster *chrna5/chrna3/chrnb4*. Further, the appropriate diagnosis of the psychiatric disease at the onset of symptoms could lead to an adequate treatment or therapy for the patient.

**Figure 2:**
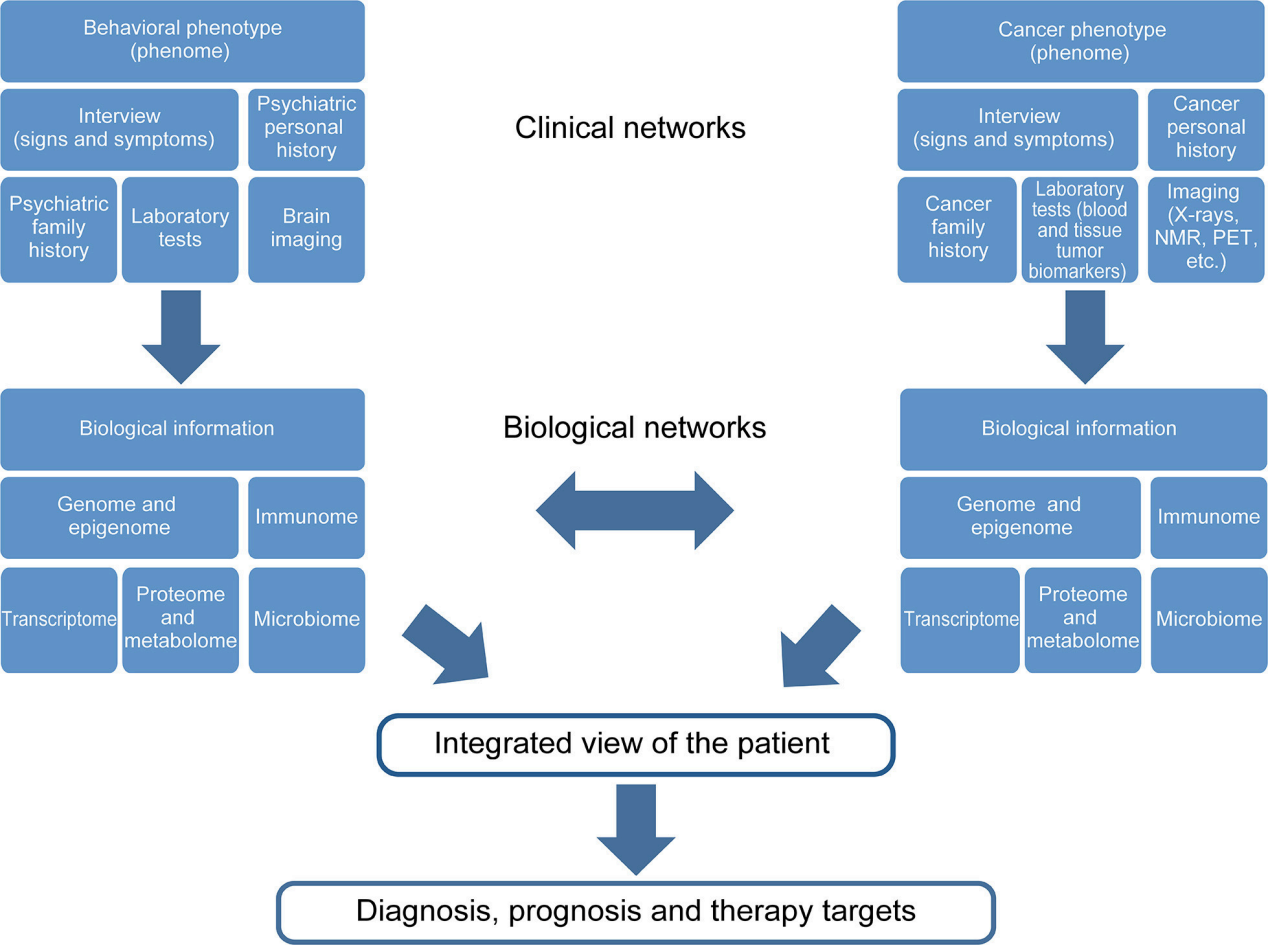
**Gestaltomics, as an integrated view of an individual, is obtained by unifying different levels of information from ranging from genetics to clinical data**. The data networks originate from different biological and clinical sources influenced by the presence of two or more diseases; such is the case of the comorbidity psychiatric disease, cancer and the social environment, which is reflected at a biological level.

During the development of psychiatric illness-cancer, the complexity of both diseases becomes increased. Thus, the global view of the individual is vital for cancer survival. Figure 2 shows a diagram describing the major levels of information regarding both psychiatric diseases and cancer implicated in the “gestaltomics” approach for disease diagnosis, prognosis, and discovery of therapeutic targets. The mechanistic view of these diseases, obtained from clinical and biological information, seems to be unified by common genetic factors leading to the activation of major biological pathways, in turn influenced by environmental factors (epigenetics), regulating the signal intensity causing several phenotypes of psychiatric illnesses as a disease spectrum (Figure 1). The task of systems biology is to unravel the complex mechanisms orchestrating such behavior. The construction of ontologies, whose principles could be applied to the systems biology of complex diseases, has been proposed in order to cope with this biological complexity.

The formation of ontologies that introduced human agents and software to organize information and execute a common goal in healthcare was proposed in 1998 (Falasconi et al., 1998; Falasconi and Stefanelli, 1998). This began with computer-based patient record (CPR) prototypes (Webster, 2001). However to achieve this goal, the problem of harmonizing data from one database to another had to be solved, this problem consisted in the definition of concepts or entities using the unification or integration of different data. The purpose of a medical ontology library is to analyze, integrate, and formalize medical terminologies of different areas or applications (Pisanelli et al., 2004), as an example, the concept of cancer can be defined from several points of view, morphological, biochemical, pathological, etiological, etc. The ontology library would serve as an informatics platform including every definition according to specified parameters. Therefore, the following principles must be followed in the construction of ontologies: (a) logical consistency (logical language and explicit formula semantics), (b) semantic coverage (all entities of its domain and all entity types of its domain), (c) modeling precision (only represents the intended models to accomplish the task of the ontology), (d) strong modularity (to organize the domain into different descriptions), and (d) scalability (the language used expresses the intended meaning according to the domain or tasks to accomplish) (Pisanelli et al., 2004).

The increasing amount of data derived from genomics led to the development of biological ontologies (Fernández-Bries et al., 2004), introducing also an integrative approach using bioinformatics (Gopalacharyulu et al., 2008). Afterwards, cognitive ontologies, based on the structure–function data from neurologically affected patients, integrated cognitive, and anatomical models and organized the cognitive components for diverse tasks into a single framework (Price and Friston, 2005). Currently, ontologies serve as “a means to standardize terminology, to enable access to domain knowledge representation, cognitive science, to verify data consistency and to facilitate integrative analysis over heterogeneous biomolecule data” (Hoehndorf et al., 2013).

The ontology proposed by the Consortium of Neuropsychiatric Phenomics continues with the sequence of platforms being implemented to improve the definition of psychiatric phenotypes through different levels or domains of knowledge (syndrome, symptom, cognitive phenome, neural systome, cellular-signalome, Proteome, genome) seeking to define a disease more accurately, including the data derived from each domain, and focusing mainly on defining the cognitive phenome of psychiatric diseases. The multivariate definition of a phenotype can lead to advances in the face of complex diseases, such as cancer and psychiatric diseases. This not only improves the definition of phenotypes but also establishes connections between intermediate phenotypes (Bilder et al., 2009a). Together with the initiative of the National Mental Health Research Domains Criteria (RDoC), it will have a direct impact on the improvement of the diagnostic taxonomy of mental disorders based on brain biology (Bilder et al., 2013).

It is interesting that, in some of the ontologies available on the web, the harmonization of different formats of bioinformatics data or reservoirs of information is being achieved. Since the principles that construct these ontologies can be applied to the bioinformatics of complex diseases, this type of initiatives from multidisciplinary groups can be a more effective approach, through Systems Biology, to address the complexity issue of diseases such as cancer and psychiatric disorders in an organized framework that would provide an integral picture of the individual and his illness.

### “Omics” studies on neuropsychiatric disorders and cancer

There few studies regarding the association of psychiatric diseases and cancer, such as schizophrenia and breast cancer (Catts et al., 2008; Bushe et al., 2009), or Alzheimer's disease with reduced risk for cancer (Roe et al., 2005), addressing a potential opportunity for biomedical research (Catalá-López et al., 2017). A promising field in “omics” studies is the association between alcohol drinking behavior and cancer.

Alcohol abuse has been recognized as a common component in different types of cancer (World Health Organization, 2014). Alcoholism is accepted as a disease and though DMS-V criteria distinguish between alcohol dependence and alcohol abuse, the diagnosis criteria is evolving. There is also a variety of phenotypes of alcoholism. Polymorphisms of the alcohol dehydrogenase (ADH1B Arg48His) and aldehyde dehydrogenase (ALDH2 Glu487Lys) genes are commonly associated with alcohol consumption and cancer.

The ADH1B gene and its alleles, Arg48His (rs1229984) and Arg370Cys (rs2066702), are associated with alcohol metabolism and drinking behavior, cancer, and human phenomes (Polimanti and Gelernter, 2017). Esophageal cancer is associated with an Arg/Arg genotype of ADH1B Arg48His, although its 48His allele has been proved to have a protective effect against this type of cancer (Mao et al., 2016). The association of ADH1B with colorectal cancer risk in Chinese population has been reported (Zhong et al., 2016). It has also been shown that this gene is correlated with gastric cancer (Chen et al., 2016). In addition, the ADH1B Arg48His allele increases lung cancer risk in carriers (Álvarez-Avellón et al., 2017). ALDH2 and ADH1B polymorphisms are associated with a higher risk for bladder cancer and alcohol abuse (Masaoka et al., 2016). Alcohol abuse also mediates the ADH1B effect on hepatitis B-related hepatocellular carcinoma risk (Liu et al., 2016), and head and neck squamous cell carcinoma (Ji et al., 2015). There are few omics studies on the field; however, a noteworthy study on the microbiome in fecal samples of alcoholic individuals could help to understand the phenotype of individuals at risk of developing colorectal cancer (Tsuruya et al., 2016).

There is yet an enormous task to be undertaken in the “omics” field of comorbidity of psychiatric diseases and cancer. The knowledge gathered from this exciting field will contribute to the successful development of personalized medical care for these patients.

## Conclusion and perspectives

The present review highlights how the vast amount of information from omics technologies in complex diseases, such as schizophrenia, present several challenges regarding data management and format harmonization of output data. Despite the challenge, some studies have performed successful analyses starting from different technological platforms (See Table 1).

**Table 1:** **Important findings in psychiatric disorders by using “omics” technologies described in this review**.

**Disease**	**Discovery according to “omics” data**	**References**
**SCHIZOPHRENIA**		
Genome	Loci 6, 8, 12, and 22 associated to schizophrenia	Combs et al., 2012
	Hundred and seventy-seven genes related to schizophrenia in brain	Glatt et al., 2005
	Allele copy number variation implicated in the development of schizophrenia	Stefansson et al., 2013
Metilome	Hypomethylation of *st6galnacl* in brain and blood	Dempster et al., 2011
Proteome	Apo1 was downregulated in CSF and RBC	Huang et al., 2007
Metabolome and lipidome	Twenty metabolites and fatty acids in serum and plasma changed in patients, changes were also observed in patients with drug treatment	Xuan et al., 2011; He et al., 2012
**AUTISM**		
Genome	ASD risk is conferred by rare variations from CNVs to SNVs	Pinto et al., 2014
	15q11.2-q13 duplications, 16p11.2 deletion, 16p11.2 duplication, and X-linked loss-of function SNVs associated to autism	
Metabolome	Changes in the levels of aminoacids in plasma, CSF, and urine. The levels of neurotransmitters and hormones are altered	Ming et al., 2012
	Succinate and glycolate in urine changed	Emond et al., 2013
Microbiome	Gut microbiota has important effects in the development of symptoms	Hsiao et al., 2013
**SUICIDE**		
Genome	The *slc6a4* gene associated to suicide in women	Gaysina et al., 2006
	*comt* gene also related to suicide	Kia-Keating et al., 2007
	*papln* and *il28ra* (rs11628713 and rs109030324) markers of suicidal ideation	Laje et al., 2009
Transcriptome	*garbrg2* expression was lower in brain of suicides	Yin et al., 2016
	Seventy-six genes for suicide are involved in neural connectivity, immune, and inflammation responses	Niculescu et al., 2015b
Proteome	CRYAB, GFAP, and SOD2 proteins expressed only in prefrontal cortex tissues from suicides	Schlicht et al., 2007

*CSF, cerebrospinal fluid; RBC, red blood cells; CNV, copy number variation; SNV, single nucleotide variation*.

Because most studies in the “omics” field are separate entities and do not integrate other levels of information, only a few have taken this approach (van Eijk et al., 2014). van Eijk et al. attempted an “omics” analysis with different levels or “layers” of genomic information (such as SNPs, methylation, and gene expression), identifying disease susceptibility loci for neuropsychiatric traits due to the enrichment of disease-specific signals when combining different genomic layers prioritizing genomic loci. This approach supported the use of whole blood for the study of brain-related diseases (van Eijk et al., 2014). This issue could be solved also for other peripheral samples through integrative studies.

Systems Biology must be able to provide proper quantitative schemes that will contribute to the understanding of underlying mechanisms and phenotype prediction in psychiatric diseases, as well as its association with other comorbid diseases such as cancer. Some groups have developed mathematical analyses using model systems exploring feasible metabolic phenotypes in human cancer cell lines and tissues (Lewis and Abder-Haleem, 2013). In this regard, a metabolic phenotype modeling performed by Diener et al. (2016) used metabolome and expression data to infer the metabolic phenotype of HeLa cancer cells. The mathematical modeling, based on the metabolite concentrations in this study, set the basis for inferring affected enzymes in a diseased state when it is not evident at genomic level. Another important advance in exploring metabolic phenotypes is the Human Metabolic Atlas database containing a set of tissue specific genome scale metabolic reconstructions of human tissues (Pornputtapong et al., 2015). Therefore, advances in multiscale modeling promises the inference of the metabolic phenotype from a cell to a whole organism. Notably, this type of studies could have the potential to improve the decision-making process regarding the type of chemotherapy administered to a cancer patient (Diener and Resendis-Antonio, 2016).

Ontologies are an excellent proposal for the integration of clinical, biological and behavioral information enabling a precise description of the disease presented by an individual. The use of multidisciplinary platforms, integrating the intermediate phenotypes contributing to the global phenotype, will provide the necessary tools for data analyses. We have already discussed the existence of different databases and software available from various platforms, which can be used to analyze experimental data derived from patient samples. We propose the development of a network derived from each type of data; the elements of such a network should be shared with the other networks of biological information. The convergence of evidence provided by bioinformatics analyses will allow the visualization of a characteristic phenotype pattern exhibited by psychiatric patients. Such evidence will lead to personalized diagnosis for each patient and, if appropriate, will also contribute to disease prognosis.

However, there is yet much work to do in order to (i) integrate clinical and “omics” data, (ii) integrate the networks from different “omics” technologies, (iii) complete data analyses from different levels of information, and (iv) compare different networks from two or more diseases affecting one individual to improve the description of his health/disease states. In this regard, the concept of “gestaltomics” will be developed by a better understanding of complex Systems Biology.

## Author contributions

NG, Research, writing, and discussion of this review; HN, Original idea, writing, discussion, and revision of this review; OR, Original idea, writing, and revision of this review.

## Funding

The present study was supported by CONACYT, Mexico. Funding code: SALUD-2015-2 No. 261516.

### Conflict of interest statement

The authors declare that the research was conducted in the absence of any commercial or financial relationships that could be construed as a potential conflict of interest.

## Abbreviations

CSF: cerebrospinal fluid
RBC: red blood cells
CNV: copy number variation
SNV: single nucleotide variation.
